# Label-free high-throughput screening assay for the identification of norepinephrine transporter (NET/SLC6A2) inhibitors

**DOI:** 10.1038/s41598-021-91700-7

**Published:** 2021-06-10

**Authors:** Hubert J. Sijben, Wieke M. van Oostveen, Peter B. R. Hartog, Laura Stucchi, Andrea Rossignoli, Giovanna Maresca, Lia Scarabottolo, Adriaan P. IJzerman, Laura H. Heitman

**Affiliations:** 1 Division of Drug Discovery and Safety, LACDR, Leiden University, P.O. Box 9502, 2300RA Leiden, The Netherlands; 2 Axxam S.p.A, Openzone Science Park, Bresso, Milan, Italy; 3 Oncode Institute, Leiden, The Netherlands

**Keywords:** Receptor pharmacology, Transporters in the nervous system, Transporters, Drug screening, High-throughput screening

## Abstract

The human norepinephrine transporter (NET) is an established drug target for a wide range of psychiatric disorders. Conventional methods that are used to functionally characterize NET inhibitors are based on the use of radiolabeled or fluorescent substrates. These methods are highly informative, but pose limitations to either high-throughput screening (HTS) adaptation or physiologically accurate representation of the endogenous uptake events. Recently, we developed a label-free functional assay based on the activation of G protein-coupled receptors by a transported substrate, termed the TRACT assay. In this study, the TRACT assay technology was applied to NET expressed in a doxycycline-inducible HEK 293 JumpIn cell line. Three endogenous substrates of NET—norepinephrine (NE), dopamine (DA) and epinephrine (EP)—were compared in the characterization of the reference NET inhibitor nisoxetine. The resulting assay, using NE as a substrate, was validated in a manual HTS set-up with a Z′ = 0.55. The inhibitory potencies of several reported NET inhibitors from the TRACT assay showed positive correlation with those from an established fluorescent substrate uptake assay. These findings demonstrate the suitability of the TRACT assay for HTS characterization and screening of NET inhibitors and provide a basis for investigation of other solute carrier transporters with label-free biosensors.

## Introduction

The uptake of neurotransmitters in and around the synaptic cleft by dedicated membrane transport proteins is a key process in the regulation of neurotransmitter signaling^1^. As such, transporter dysfunction and aberrant levels of synaptic neurotransmitters have been linked to the manifestation of an array of psychiatric disorders including depression, anxiety and attention-deficit hyperactive-disorder^2^. Monoamine transporters of the solute carrier transporter family 6 (SLC6) mediate the rapid clearance of released monoamine neurotransmitters (e.g., dopamine, norepinephrine, serotonin) and are therefore considered major drug targets for the aforementioned psychiatric disorders^3,4^.

The norepinephrine transporter (NET, SLC6A2) facilitates sodium- and chloride-dependent uptake of norepinephrine, with overlapping substrate specificity for dopamine and epinephrine^1,5^. NET is mainly expressed at synaptic terminals in the nervous system where it is a regulator of noradrenergic signaling, which affects physiological processes such as mood, behavior, heart rate and blood pressure^6^. Currently, NET is a therapeutic target of tricyclic antidepressants (TCAs), selective norepinephrine reuptake inhibitors (NRIs) and serotonin-norepinephrine reuptake inhibitors (SNRIs) for the treatment of depression^2,3,7^. In addition, NET is targeted by radiolabeled “theranostics” for imaging and treatment of neuroendocrine tumors^8,9^, as well as drugs of abuse (e.g., cocaine, amphetamine) which are known to inhibit NET^3,10^. The continuing clinical use of (S)NRIs to treat anxiety and depression as well as the recent developments of NET inhibitors as potential treatment for incontinence^11^, excessive sleepiness^12^ and neuropathic pain^13,14^ still warrant the discovery of novel NET inhibitors.

Conventionally, in vitro methods to functionally characterize NET inhibitors are based on inhibition of uptake of a radiolabeled substrate (e.g., [^3^H]norepinephrine^15,16^) or a fluorescent substrate^17,18^. While these methods generally provide reliable IC_50_ values for NET inhibitors, the use of labeled substrates has practical downsides such as high costs, waste management, safety precautions and availability of suitable (fluorescent) substrates, limiting the broad implementation of these principles for drug screening^19^. Recently, our group developed a novel functional ‘transport activity through receptor activation’ (TRACT) assay based on a label-free impedance-based technology for the equilibrative nucleoside transporter 1 (ENT1, SLC29A1)^20,21^ and the dopamine transporter (DAT, SLC6A3)^22^. In this bioassay a transporter that shares its substrate (e.g., adenosine, dopamine) with a G protein-coupled receptor (GPCR) is expressed in live cells together with a cognate GPCR. Uptake of substrate by the transporter decreases its local extracellular concentration, thereby limiting the ability of the substrate to activate the GPCR. Conversely, pharmacological inhibition of the transporter augments the substrate-induced GPCR response, providing an assay window for identification of transporter modulators. So far, the TRACT assay principle has been demonstrated in a low-throughput setting, while the screening potential of the assay has not yet been investigated.

In this study, a label-free TRACT assay was developed and validated for the human NET using an impedance-based biosensor, xCELLigence^23–25^. To develop the assay we used a modified human embryonic kidney (HEK) 293 cell line with doxycycline-inducible overexpression of NET and endogenous expression of adrenergic receptors (JumpIn-NET). Endogenous substrates of NET (norepinephrine, dopamine and epinephrine) were used to explore the substrate specificity of NET inhibitors and to maximize the assay window. Following optimization, the assay was validated in a manual 96-well high-throughput screening (HTS) set-up, demonstrating an “excellent assay” window, according to definition by Zhang^26^. Several reference NET inhibitors were tested for their inhibitory potencies, which showed a strong correlation with potencies from an established fluorescent substrate uptake assay^18^. These results render the TRACT assay suitable for characterization of NET inhibitors and demonstrate that the assay is amenable to HTS. The detailed read-out, physiological setting and label-free nature of the method make the TRACT assay a meaningful alternative to conventional label-based assays for SLCs.

## Materials and methods

### Chemicals and reagents

Jump-In T-REx HEK 293 cells modified for doxycycline-inducible overexpression of the wild-type human norepinephrine transporter (JumpIn-NET) were provided by CeMM (Research Center for Molecular Medicine, Medical University of Vienna, Austria). These cells were used in the TRACT and the fluorescent substrate uptake assays to allow a good comparison. Benztropine mesylate, desipramine hydrochloride, Dulbecco’s Modified Eagle’s Medium (DMEM), dopamine hydrochloride, doxycycline hyclate, (−)-epinephrine (+)-bitartrate salt, L-(−)-norepinephrine (+)-bitartrate salt monohydrate and (±)-propranolol hydrochloride were purchased from Sigma Aldrich (St. Louis, MO, USA). Nisoxetine hydrochloride, maprotiline hydrochloride and yohimbine hydrochloride were purchased from Santa Cruz Biotechnology (Dallas, TX, USA). GBR12909 dihydrochloride and reboxetine mesylate were purchased from Toronto Research Chemicals (Toronto, ON, Canada). Amitriptyline hydrochloride, atomoxetine hydrochloride, bupropion hydrochloride, milnacipran hydrochloride and nortriptyline hydrochloride were purchased from Tebu-Bio (Heerhugowaard, The Netherlands). Cocaine hydrochloride was purchased from Duchefa Farma (Haarlem, The Netherlands), where Leiden University has been certified for its use in pharmacological experiments. All other chemicals were of analytical grade and obtained from standard commercial sources.

### JumpIn-NET cell line generation

Jump-In T-REx HEK 293 (JumpIn) cells were cultured and transfected according to the manufacturer’s protocol (ThermoFisher Scientific) and as described previously^22^. Cells were split twice a week in growth medium containing 200 µg/ml hygromycin B and 5 µg/ml blasticidin. For transfection, a codon optimized ORF (Addgene #131891) for the human norepinephrine transporter (SLC6A2, ORF: NM_001043.3) was cloned into a gateway-compatible pJTI R4 DEST CMV TO pA expression vector. This allows doxycycline (dox)-inducible expression of NET in successfully transfected cells. Cells were selected in medium containing 2 mg/ml geneticin (G418) and 5 μg/ml blasticidin for 2 to 4 weeks and resistant clones were pooled for use in all further experiments.

### Cell culture

JumpIn-NET cells were grown as adherent cells in high glucose DMEM supplemented with 10% (v/v) fetal calf serum (FCS), 2 mM Glutamax, 100 IU/ml penicillin and 100 µg/ml streptomycin (culture medium) at 37 °C and 7% CO_2_. Upon thawing, cells were cultured in regular culture medium for 1–2 passages. Then, cells were cultured up to one week in culture medium supplemented with 2 mg/ml G418 and 5 µg/ml blasticidin to select only the transfected clones. Cells were subsequently switched to regular culture medium, waiting at least 24 h before performing an experiment. Cell cultures were split twice a week at ratios of 1:8–1:16 in 10 cm plates.

### TRACT assays

Label-free TRACT assays were performed using the xCELLigence real-time cell analysis (RTCA) technology as reported previously^22^. Impedance values, which are measured continuously at a frequency of 10 kHz, for each well are converted by the RTCA software to the dimensionless parameter Cell Index (CI) using the following formula:

$$CI = \frac{{\left( {Z_{i} - Z_{0} } \right){\Omega }}}{{15{\Omega }}}$$

where Z_i_ is the impedance at any given time point and Z_0_ is the baseline impedance that is measured at the start of each experiment^27^.

Assays were performed at 37 °C and 5% CO_2_ in 96-well E-plates in a final volume of 100 μl per well. Background impedance was measured in 45 μl (one compound addition) or 40 μl (two compound additions) culture medium prior to cell seeding. Compounds were added in 5 μl per addition using a VIAFLO 96 handheld electronic 96 channel pipette (INTEGRA Biosciences, Tokyo, Japan). All conditions were tested in duplicate on each plate.

#### Cell preparation and monitoring

JumpIn-NET cells were grown to 70–80% confluence on the day of the experiment. Baseline impedance was measured using culture medium with or without 1 μg/ml doxycycline (dox). Cells were seeded in E-plates at 60,000 cells/well in culture medium. Cells were left to sink to the bottom for 30 min at room temperature before placing the E-plate in the recording station at 37 °C. Cells were left to grow overnight for 22–24 h while recording impedance every 15 min.

#### Cell pretreatment

In GPCR antagonist experiments, cells were pretreated by the addition of a single concentration (1 μM) of yohimbine (alpha-2 adrenergic receptor antagonist), propranolol (non-selective beta adrenergic receptor antagonist) or a 1:1 mix of both antagonists in DMSO. In TRACT assays, cells were pretreated with either a single concentration (1 μM) of nisoxetine (high affinity NET inhibitor^28^) or six increasing concentrations of a NET inhibitor. Due to strict local regulations, cocaine could only be tested in the TRACT assay and not in the fluorescent substrate uptake assay. For all pretreatments DMSO was kept at 0.1% per well and impedance was measured for 1 h prior to substrate addition.

#### Cell stimulation

Cells with or without pretreatment were stimulated by the addition of either norepinephrine (NE), dopamine (DA) or epinephrine (EP) as a substrate dissolved in 1 mM ascorbic acid in PBS. Note, ascorbic acid was used as an antioxidant for the monoamine neurotransmitter substrates^29^. In antagonist experiments cells were stimulated with a submaximal concentration (EC_80_) of substrate (i.e. 10 μM NE; 100 μM DA; 10 μM EP). In TRACT assays cells were stimulated with seven increasing concentrations of substrate to determine substrate potency. To determine the inhibitory potencies of NET inhibitors, cells were stimulated with a submaximal (EC_20_) concentration of substrate (i.e. 1 μM NE; 3.16 μM DA; 1 μM EP). For a total of 30 min after stimulation, impedance was measured initially every 15 s for 25 min and then every minute.

### TRACT assay HTS validation

The TRACT assay was assessed for reproducibility, robustness and amenability to high-throughput screening (HTS) according to methods described previously in assay guidance manuals^30^. Three 96-well E-plates were run consecutively per day on three separate days. Cells were induced with 1 µg/ml dox at the start of each experimental run. After cell seeding, E-plates were left at room temperature for 30 min and subsequently placed inside an incubator for 22 h. Each E-plate had an alternating interleaved layout consisting of high, mid and low signals for which cells were pretreated with either 1 µM nisoxetine, 10 nM nisoxetine or vehicle (DMSO), respectively. After 1 h pretreatment, all wells were stimulated with a submaximal (EC_20_) concentration (1 µM) of NE. Impedance was recorded for 30 min after substrate addition. Immediately after recording the NE response the next E-plate was inserted in the RTCA recording station. Compound additions were done using a VIAFLO 96 handheld electronic 96 channel pipette. All other handlings were performed manually.

### Fluorescent substrate uptake assay

Fluorescent substrate uptake assays were performed using the Neurotransmitter Transporter Uptake Assay Kit (Molecular Devices, San Jose, CA, USA) following the supplier’s protocol. JumpIn-NET cells were seeded at 20,000 cells/well in culture medium in presence of 1 μg/ml dox in clear-bottom, black-walled 384 microtiter plates pre-coated with poly-D-lysine (Twin Helix, Milan, Italy) at 37 °C and 5% CO_2_. After 24 h medium was removed and 20 μl/well of Standard Tyrode’s buffer (130 mM NaCl, 5 mM KCl, 2 mM CaCl_2_, 1 mM MgCl_2_, 5 mM NaHCO_3_, 20 mM HEPES, pH 7.4) was added. Cells were pretreated by addition of 10 μl/well NET inhibitor (increasing concentrations), inhibitor control (30 μM desipramine) or vehicle control (buffer only) in Standard Tyrode’s buffer at 0.1% DMSO (final concentration) for 1 h. Subsequently, cells were treated with 15 μl/well loading dye solution in Standard Tyrode’s buffer. Cells were incubated for 1 h, after which the fluorescence was measured for 60 s using a FLIPR^TETRA^ plate reader (Molecular Devices, San Jose, CA, USA). All conditions were tested in quadruplicate on each plate except the vehicle and inhibitor controls, which each had 16 replicates per plate.

### Data analysis

#### TRACT assay

Experimental data was recorded using RTCA Software v2.0 or v2.1.1 (ACEA Biosciences). For analysis of substrate-induced responses CI values were normalized to the time point prior to substrate addition to obtain normalized CI (nCI) values. Data were exported from RTCA Software and all subsequent analyses were performed in GraphPad Prism v8.1.1 (GraphPad Software, San Diego, CA, USA). The nCI values of vehicle-only controls were subtracted from all other data points to baseline-correct for any substrate-independent effects. Substrate-induced responses were quantified by taking the net area under the curve (AUC) of the first 30 min after substrate addition. The apparent potency values of NET substrates and the inhibitory potency values of NET inhibitors were obtained by fitting the AUC data with non-linear regression to a sigmoidal concentration-effect curve with a variable pseudo-Hill slope.

#### TRACT assay HTS validation

For intra-plate variability tests, the net AUC of non-corrected nCI values were used to determine the signal window (SW, indicating dynamic range of the signal) using the following formula^30^:

$$SW = \frac{{\left( {AVG_{high} - \frac{{3SD_{high} }}{\sqrt n }} \right) - \left( {AVG_{low} + \frac{{3SD_{low} }}{\sqrt n }} \right)}}{{\frac{{SD_{high} }}{\sqrt n }}}$$

where *n* is the number of technical replicates per compound in the intended screening assay (e.g., for duplicate measurements *n* = 2), AVG is the average and SD is the standard deviation of the AUC of the high or low signal. Similarly, the Z′ factor (Z′, indicating separation of the high and low signals) is calculated using the following formula^26,30^:

$$Z^{\prime } = \frac{{\left( {AVG_{high} - \frac{{3SD_{high} }}{\sqrt n }} \right) - \left( {AVG_{low} + \frac{{3SD_{low} }}{\sqrt n }} \right)}}{{\left( {AVG_{high} - AVG_{\min } } \right)}}$$

The reported SW and Z′ are the mean ± SEM of all nine E-plates. According to Iversen et al*.*^30^, the recommended acceptance criterion for an HTS amenable assay is a SW ≥ 2 or Z′ ≥ 0.4.

#### Fluorescent substrate uptake assay

Fluorescence data was collected using the FLIPR^TETRA^ plate reader. The fluorescent substrate uptake was quantified by taking the AUC over 60 s of the fluorescence signals that were recorded 1 h after addition of the loading dye solution. The AUC values were normalized to percentage activity by the following formula:

$$\% activity = \frac{X - VC}{{IC - VC}} \times - 100\%$$

where X is the AUC of the tested condition, VC is the AUC of the vehicle control (buffer only) and IC is the AUC of the inhibitor control (30 μM desipramine). Here, a negative value of -100% indicates complete inhibition of NET. The inhibitory potency values of NET inhibitors were obtained in Genedata Screener software v16.0.6 (Genedata, Basel, Switzerland) by fitting the normalized activity data with non-linear regression to a sigmoidal concentration-effect curve with a variable pseudo-Hill slope.

#### Statistics

Data are shown as mean ± standard error of the mean (SEM) of at least three separate experiments each performed in duplicate, unless stated otherwise. Significant difference between two mean potency values was determined by an unpaired two-tailed Student’s t-test. Significant difference between the mean potencies found in two assays was determined by a paired two-tailed Student’s t-test. Comparison of multiple mean values to each other or a vehicle control was done using a one-way ANOVA with Tukey’s post-hoc test or Dunnett’s post-hoc test, respectively. Differences were considered statistically significant when p-values were below 0.05.

## Results

### Presence of NET attenuates substrate-induced cellular responses

In order to detect NET function in a label-free TRACT assay, a JumpIn cell line with dox-inducible expression of NET (JumpIn-NET) was generated. Suitable substrates for the TRACT assay were selected based on the criteria that the substrate should both be transported by NET and activate the cognate GPCR. Besides norepinephrine (NE), which is the most common endogenous substrate of NET, there are at least two other endogenous substrates known to be transported by NET and act as GPCR agonists, namely dopamine (DA) and epinephrine (EP). To evaluate which substrate was the most applicable for use in the TRACT assay for NET, all three substrates were extensively assessed for their ability to induce a cellular response on the JumpIn-NET cells and for their suitability to characterize the reference NET inhibitor nisoxetine. In the following sections we describe the results for each of the three substrates separately.

#### Norepinephrine (NE)

**Figure 1:**
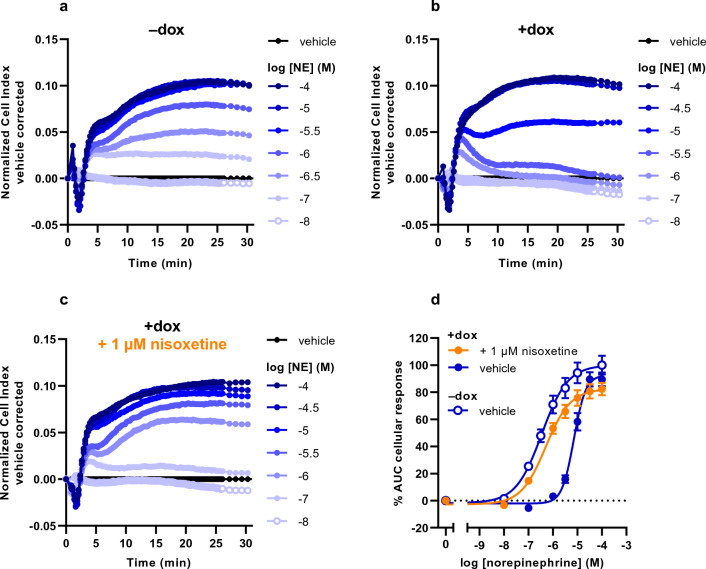
Characterization of the norepinephrine (NE) response on JumpIn-NET cells in the TRACT assay. Representative vehicle-corrected normalized Cell Index traces of vehicle-pretreated JumpIn-NET cells (**a**) in the absence (−dox) or (**b**) in the presence of 1 µg/ml dox (+dox). (**c**) Dox-treated cells were pretreated for 1 h with 1 µM nisoxetine prior to stimulation with NE. (**d**) Combined concentration-effect curves of NE on vehicle- or dox-treated JumpIn-NET cells. Cellular response is expressed as the net AUC of the first 30 min after stimulation. Data are normalized to the response of 100 µM NE on vehicle-treated (–dox) cells. Data are shown as the mean ± SEM of three to eight individual experiments each performed in duplicate.

To assess the substrate-induced cellular response in cells without and with NET in the TRACT assay, JumpIn-NET cells were cultured for 22–24 h in E-plates in the absence (vehicle-treated, ‒dox) or presence (dox-treated, + dox) of 1 μg/ml dox, respectively. Cells were pretreated for 1 h with vehicle prior to stimulation with increasing concentrations of substrate. Upon stimulation of vehicle-treated cells with NE, the vehicle-corrected normalized Cell Index (nCI) transiently decreased within the first 2 min after stimulation followed initially by a rapid ascent of the nCI to a peak around 5 min and then a more prolonged increase in nCI leading to a plateau between 20 and 30 min (Fig. 1a). In dox-treated cells, NE induced a comparable response in the first 4 min after stimulation, however, at NE concentrations at or below 10 µM the nCI stabilized or gradually decreased back to baseline after 30 min (Fig. 1b). The apparent potency of NE was 18-fold lower and the pseudo-Hill slope was more than twofold higher in dox-treated cells (pEC_50_ = 5.2 ± 0.1, n_H_ = 2.1 ± 0.2) compared to vehicle-treated cells (pEC_50_ = 6.4 ± 0.1, n_H_ = 0.8 ± 0.1), indicating the presence of NET leads to removal of extracellular NE (Fig. 1d; Table 1).

**Table 1:** Apparent potency values (pEC_50_) and pseudo-Hill slopes (n_H_) of norepinephrine (NE), dopamine (DA) and epinephrine (EP) in the absence (−) or presence of 1 µM nisoxetine on JumpIn-NET cells treated with vehicle (−dox) or doxycycline (+dox) in TRACT assays.

Substrate		Pretreatment	pEC_50_ ± SEM^a,b^	Slope (n_H_) ± SEM^a,b^	*n*
NE	−dox	–	6.4 ± 0.1	0.8 ± 0.1	7
	−dox	Nisoxetine	6.5 ± 0.0	0.9 ± 0.1	5
	+dox	–	5.2 ± 0.1***	2.1 ± 0.2***	8
	+dox	Nisoxetine	6.3 ± 0.1^d^	0.9 ± 0.1^d^	6
DA	−dox	–	5.1 ± 0.1	0.8 ± 0.1	3
	+dox	–	4.7 ± 0.0**	1.5 ± 0.1**	7
	+dox	Nisoxetine	4.4 ± 0.1^c^	0.6 ± 0.0^d^	4
EP	−dox	–	6.4 ± 0.2	0.6 ± 0.0	3
	+dox	–	5.4 ± 0.1***	0.8 ± 0.1	6
	+dox	Nisoxetine	6.3 ± 0.1^d^	0.6 ± 0.1	3

^a^pEC_50_ values and pseudo-Hill slopes are reported as the mean ± SEM of three to eight individual experiments performed in duplicate.

^b^Significant difference between two mean potency values was determined by an unpaired two-tailed Student’s t-test. **p < 0.01, ***p < 0.001, compared to vehicle-treated (− dox) cells in the absence (–) of 1 µM nisoxetine using the same substrate. ^c^*p* < 0.05, ^d^*p* < 0.001, compared to doxycycline-treated (+dox) cells in the absence (–) of 1 µM nisoxetine using the same substrate.

#### Dopamine (DA)

**Figure 2:**
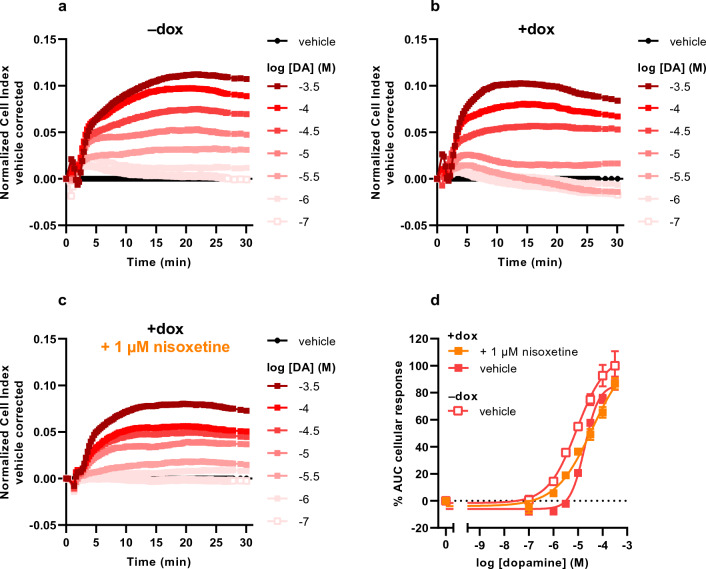
Characterization of the dopamine (DA) response on JumpIn-NET cells in the TRACT assay. Representative vehicle-corrected normalized Cell Index traces of vehicle-pretreated JumpIn-NET cells (**a**) in the absence (−dox) or (**b**) in the presence of 1 µg/ml dox (+dox). (**c**) Dox-treated cells were pretreated for 1 h with 1 µM nisoxetine prior to stimulation with DA. (**d**) Combined concentration-effect curves of DA on vehicle- or dox-treated JumpIn-NET cells. Cellular response is expressed as the net AUC of the first 30 min after stimulation. Data are normalized to the response of 316 µM DA on vehicle-treated (–dox) cells. Data are shown as the mean ± SEM of three to seven individual experiments each performed in duplicate.

Stimulation of vehicle-treated cells with DA resulted in an initial rapid increase in nCI peaking at 5 min followed by a more gradual increase until a plateau was reached between 15 and 30 min (Fig. 2a). In dox-treated cells, the magnitude and extent of DA responses at concentrations greater than 10 μM was similar to vehicle-treated cells (Fig. 2b). However, at DA concentrations of 10 μM and lower the nCI slightly peaked at 5 min and gradually returned to baseline within 30 min. In contrast to NE, the apparent potency of DA was only twofold lower with an increased pseudo-Hill slope in dox-treated cells (pEC_50_ = 4.7 ± 0.0, n_H_ = 1.5 ± 0.1) compared to vehicle-treated cells (pEC_50_ = 5.1 ± 0.1, n_H_ = 0.8 ± 0.1) (Fig. 2d, Table 1). This indicates that DA is less potent than NE on JumpIn-NET cells and that the presence of NET leads to a slight potency shift of DA.

#### Epinephrine (EP)

**Figure 3:**
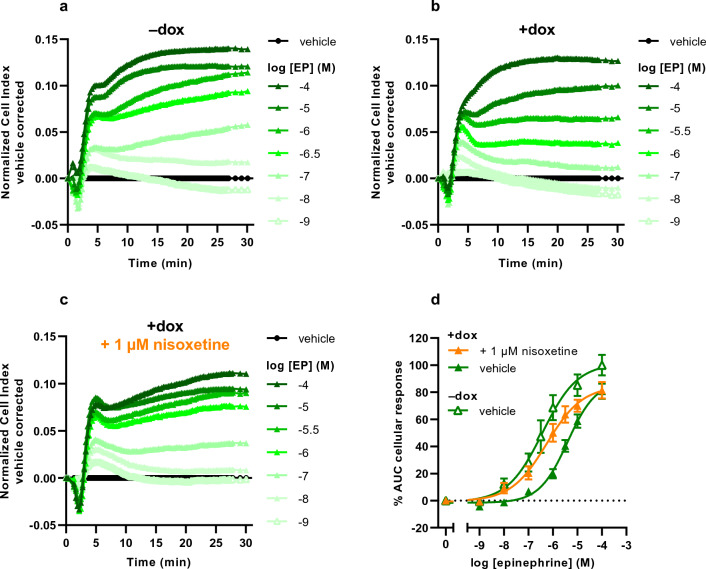
Characterization of the epinephrine (EP) response on JumpIn-NET cells in the TRACT assay. Representative vehicle-corrected normalized Cell Index traces of vehicle-pretreated JumpIn-NET cells (**a**) in the absence (−dox) or (**b**) in the presence of 1 µg/ml dox (+dox). (**c**) Dox-treated cells were pretreated for 1 h with 1 µM nisoxetine prior to stimulation with EP. (**d**) Combined concentration-effect curves of EP on vehicle- or dox-treated JumpIn-NET cells. Cellular response is expressed as the net AUC of the first 30 min after stimulation. Data are normalized to the response of 100 µM EP on vehicle-treated (−dox) cells. Data are shown as the mean ± SEM of three to six individual experiments each performed in duplicate.

Addition of EP to vehicle-treated JumpIn-NET cells lead to a brief decrease in nCI in the first 2 min after stimulation, then a sharp increase in nCI that peaked at 4 min (Fig. 3a). The rise in nCI then temporarily halted before gradually surging to a plateau within 30 min. Cells pretreated with dox demonstrated a similar response to EP within the first 4 min (Fig. 3b). At concentrations of EP lower than 10 μM the nCI briefly dropped followed by a plateau or a steady decline back to baseline between 7 and 30 min. The apparent potency of EP was decreased tenfold with no change in the pseudo-Hill slope in dox-treated cells (pEC_50_ = 5.4 ± 0.1, n_H_ = 0.8 ± 0.1) compared to vehicle-treated cells (pEC_50_ = 6.4 ± 0.2, n_H_ = 0.6 ± 0.0), indicating that extracellular levels of EP are lowered in the presence of NET.

### Substrate-induced responses are mainly caused by alpha-2 receptor activation

**Figure 4:**
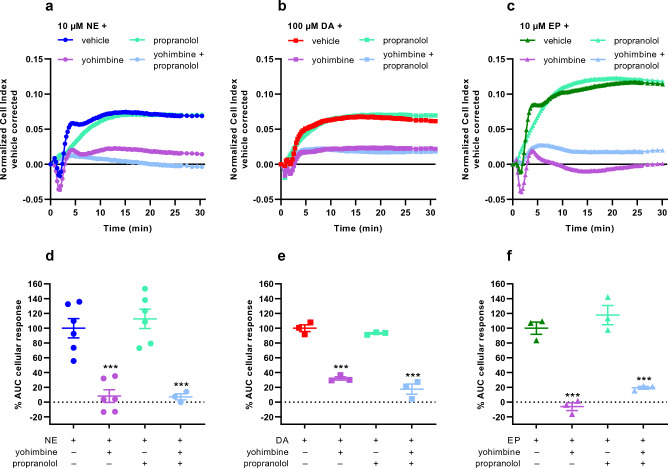
Assessment of GPCR involvement in substrate-specific cellular responses on dox-treated JumpIn-NET cells. Prior to stimulation with substrate, dox-treated cells were pretreated for 1 h with vehicle or 1 µM antagonist for α_2_ARs (yohimbine; purple) or βARs (propranolol; mint green) or a 1:1 mixture of both antagonists (light blue). Subsequently, cells were stimulated with an EC_80_ of either (**a**,**d**) norepinephrine (NE, 10 µM; circle), (**b**,**e**) dopamine (DA, 100 µM; square), or (**c**,**f**) epinephrine (EP, 10 µM; triangle). (**a**–**c**) Representative vehicle-corrected normalized Cell Index traces of substrate-induced cellular responses. (**d**–**f**) Net AUC of the first 30 min after stimulation. Data are normalized to the response of the respective substrate (100%) in vehicle-pretreated cells. Data are shown as mean ± SEM of three to six individual experiments each performed in duplicate. Comparison of multiple mean values to vehicle control was done using a one-way ANOVA with Dunnett’s post-hoc test. *** p < 0.001 compared to substrate only.

To validate whether activation of GPCRs on JumpIn-NET cells was related to substrate-induced cellular responses in the TRACT assay, dox-treated cells were pretreated for 1 h with two different monoamine GPCR antagonists prior to stimulation with a submaximal concentration (EC_80_) of substrate (Fig. 4). Involvement of alpha-2 adrenergic receptors (α_2_ARs) was assessed in the presence of 1 μM yohimbine, while 1 μM propranolol was used as a non-selective beta adrenergic receptor (βAR) antagonist. In the presence of yohimbine, the overall NE-induced cellular response significantly decreased (*p* < 0.001) by 92% compared to vehicle-pretreated cells (Fig. 4a, d). The transient decrease in nCI 2 min after NE stimulation was more negative in the presence of yohimbine, but the response kinetics of this part of the trace were not altered (Fig. 4a). However, this negative peak did not occur when cells were pretreated with propranolol, suggesting that this early-phase response is βAR-mediated. To assess the contribution of βAR activation to the overall NE-induced cellular response, yohimbine and propranolol were used simultaneously. Dual antagonist pretreatment prevented the early negative peak response, but did not cause a further decrease in the overall cellular response (Fig. 4a, d).

When dox-treated cells were stimulated with DA, yohimbine was able to significantly reduce (*p* < 0.001) the DA-induced response by 68% compared to vehicle-pretreated cells (Fig. 4b, e). Neither propranolol alone nor a combination of yohimbine and propranolol could further reduce the cellular response.

Similar to NE, pretreatment with yohimbine significantly reduced (*p* < 0.001) the overall EP-induced cellular response compared to vehicle-pretreated cells, whereas the rapid nCI decrease in the first 2 min after stimulation was more negative (Fig. 4c, f). In the presence of the βAR antagonist the initial negative peak was abolished, but the overall cellular response was not affected. Pretreatment with both yohimbine and propranolol slightly elevated the cellular response compared to yohimbine alone, albeit not significantly (*p* = 0.203). Overall, these data demonstrate that the substrates used in this study mainly exert their observed effects in the TRACT assay through activation of α_2_ARs.

### Nisoxetine rescues apparent potency of NE and EP, but not DA

To determine whether pharmacological inhibition of NET leads to altered substrate responsiveness in the TRACT assay, dox-treated JumpIn-NET cells were pretreated for 1 h with the selective NET inhibitor nisoxetine (1 μM, final concentration) prior to addition of increasing concentrations of substrate. Addition of 1 μM nisoxetine itself did not affect the nCI during the 1 h pretreatment (Supplementary Fig. S1j). Moreover, nisoxetine pretreatment did not change the apparent potency of NE in vehicle-treated cells (pEC_50_ = 6.5 ± 0.0, n_H_ = 0.9 ± 0.1; Table 1), which demonstrates that NE signaling is not affected by nisoxetine in the absence of NET. In dox-treated cells, stimulation with NE after nisoxetine pretreatment generated nCI traces with a shape comparable to those observed in vehicle-treated cells (compare Fig. 1c and a, respectively), resulting in a complete restoration of the pseudo-Hill slope and apparent potency of NE (pEC_50_ = 6.3 ± 0.1, n_H_ = 0.9 ± 0.1; Fig. 1d, Table 1). A similar trend was observed in dox-treated cells stimulated with EP (compare Fig. 3c and a, respectively), for which the apparent potency of EP was significantly (p = 0.0008) enhanced in the presence of nisoxetine (pEC_50_ = 6.3 ± 0.1, n_H_ = 0.6 ± 0.1; Fig. 3d, Table 1) compared to vehicle-pretreated cells. Interestingly, pretreatment of dox-treated cells with nisoxetine enhanced DA responses at concentrations of 10 μM or less, but not at higher DA concentrations (Fig. 2c), resulting in a slight decrease in apparent potency of DA and a decreased pseudo-Hill slope (pEC_50_ = 4.4 ± 0.1, n_H_ = 0.6 ± 0.0; Fig. 2d, Table 1). This suggests the magnitude and extent of the restoration of substrate-induced cellular responses by pharmacological inhibition of NET depends on the substrate used.

### NE provides the largest assay window for the determination of NET inhibitor potency

**Figure 5:**
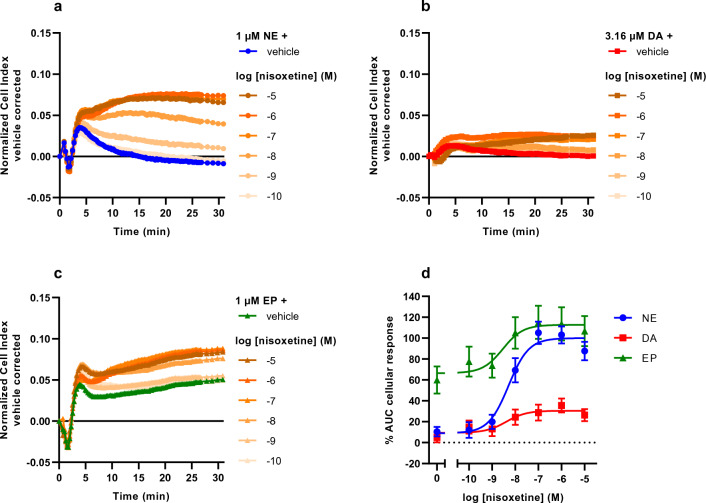
Nisoxetine potentiates substrate-induced cellular response in dox-treated JumpIn-NET cells in the TRACT assay. Representative vehicle-corrected normalized Cell Index traces are shown of dox-treated cells pretreated for 1 h with increasing concentrations of nisoxetine (orange), stimulated with a submaximal (EC_20_) concentration of (**a**) norepinephrine (NE, 1 µM; circle), (**b**) dopamine (DA, 3.16 µM; square), or (**c**) epinephrine (EP, 1 µM; triangle). (**d**) Combined concentration-inhibition curve of nisoxetine upon stimulation with either NE, DA or EP. Cellular response is expressed as the net AUC of the first 30 min after stimulation. To demonstrate the assay window for each substrate, data are normalized to the response of 1 µM NE in the presence of 1 µM nisoxetine (set at 100%) and the vehicle response (set at 0%). Data are shown as mean ± SEM of four to seven individual experiments performed in duplicate.

The main purpose of the TRACT assay is to identify transporter inhibitors and, subsequently, determine their inhibitory potency (IC_50_) values. After characterization of the various substrate-induced cellular responses in JumpIn-NET cells, we assessed which substrate was most suitable to determine the inhibitory potencies of NET inhibitors in the TRACT assay (Fig. 5). Dox-treated cells were pretreated for 1 h with increasing concentrations of the reference NET inhibitor nisoxetine and subsequently stimulated with a submaximal concentration of substrate. For each substrate, a submaximal concentration (EC_20_) was selected as this resulted in the largest possible assay window to detect NET inhibition.

Nisoxetine was able to dose-dependently augment the cellular response for all three substrates (Fig. 5). However, the largest assay window, i.e., the relative difference between the vehicle response and the maximum response was obtained with NE as a substrate (Fig. 5a, d). Specifically, nisoxetine enhanced the cellular response of EP to the same maximum as when NE was used (Fig. 5c, d), but since the basal response of EP in vehicle-pretreated cells was approximately 60% higher than that of NE this resulted in a smaller assay window compared to NE. With DA as a substrate the maximum enhancement in cellular response that was attained within the concentration range of nisoxetine was roughly 25% of the maximum NE response, resulting in the least favorable assay window (Fig. 5b, d). The IC_50_ values for nisoxetine that were determined in the TRACT assay using the various substrates (NE: pIC_50_ = 8.3 ± 0.1, DA: pIC_50_ = 8.4 ± 0.3, EP: pIC_50_ = 8.8 ± 0.3) were not significantly different from each other (*p* = 0.37), showing that the inhibitory potency of nisoxetine was not dependent on the type of substrate used.

### HTS assay validation and comparison with an orthogonal assay

**Figure 6:**
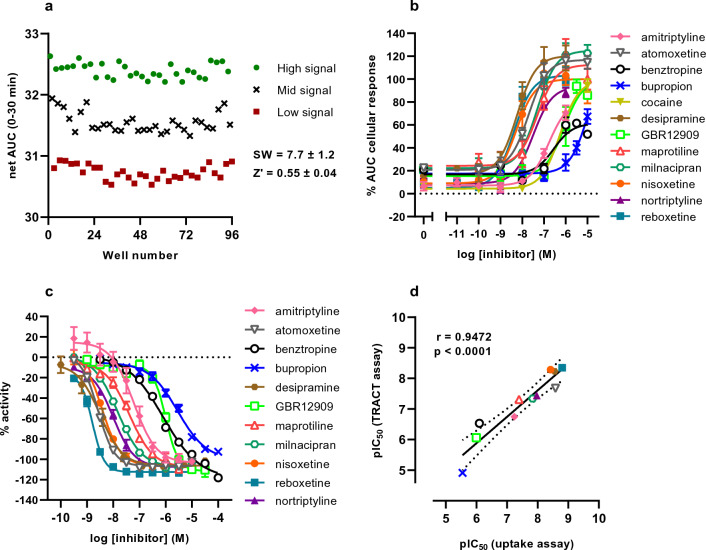
TRACT assay HTS validation and characterization of NET inhibitors in the TRACT assay and the fluorescent substrate uptake assay. (**a**) Validation of the reproducibility and robustness of the TRACT assay. Representative graph of a 96-well E-plate (row-oriented) with wells producing high (1 μM nisoxetine + 1 μM NE), mid (10 nM nisoxetine + 1 μM NE) and low (vehicle + 1 μM NE) cellular responses. Data is presented as the net AUC of the raw nCI traces of the first 30 min after NE stimulation. Each data point in the graph represents a single well. Signal window (SW) and Z′ factor (Z′) are shown as mean ± S.E.M. and were calculated according to the formulas in TRACT assay HTS validation section. (**b**) Concentration-inhibition curves of NET inhibitors in the TRACT assay. Cells are pretreated for 1 h with vehicle or one of six increasing concentrations of inhibitor, then stimulated with 1 µM NE or vehicle. Data were normalized to the average top and bottom values of the nisoxetine concentration-inhibition curve. Data are shown as mean ± SEM of three to seven individual experiments each performed in duplicate. (**c**) Concentration-inhibition curves of NET inhibitors in the fluorescent substrate uptake assay. Cells were pretreated for 1 h with vehicle or one of ten increasing concentrations of inhibitor, followed by addition of loading dye solution for 1 h. Data were normalized according to the formula in Fluorescent substrate uptake assay section. Data are shown as the mean ± SEM of three to five individual experiments each performed in quadruplicate. (**d**) Correlation plot between pIC_50_ values obtained in the TRACT assay and the fluorescent substrate uptake assay. The Pearson’s r coefficient and the corresponding *p*-value of the linear correlation (solid line) are shown. The dotted line represents the 95% confidence interval.

After defining the optimal assay conditions, the TRACT assay was assessed and validated for its high-throughput screening (HTS) compatibility. Reproducibility and robustness of the assay window were assessed by running three individual 96 E-plates each day for three consecutive days. In this test run E-plates comprised of wells in an interleaved format producing high (1 μM nisoxetine + 1 μM NE), mid (10 nM nisoxetine + 1 μM NE) and low (vehicle + 1 μM NE) cellular responses. The test run resulted in a signal window (SW) of 7.7 ± 1.2 and a Z’ factor (Z’) of 0.55 ± 0.04 (Fig. 6a).

Next, we determined the inhibitory potency of several reference NET inhibitors in the TRACT assay (Fig. 6b, Table 2) and in an orthogonal fluorescent substrate uptake assay (Fig. 6c, Table 2). The inhibitors were selected to represent a wide range of inhibitory potencies on NET. Due to strict local regulations cocaine could only be assessed in the TRACT assay; likewise amphetamines could not be tested in either of the two assays. In the TRACT assay the inhibitors generally did not affect the nCI on their own during the 1 h pretreatment (Supplementary Fig. S1). Upon stimulation with 1 µM NE, all inhibitors demonstrated dose-dependent enhancement of the NE response indicating inhibition of NET (Fig. 6b). Inhibitory potencies of NET inhibitors in the TRACT assay ranged over more than 3 log-units (Table 2). Moreover, a strong correlation (Pearson’s r = 0.9472, *p* < 0.0001) was observed when comparing the pIC_50_ values to a more conventional fluorescent substrate uptake assay (Fig. 6d). Of note, the TRACT assay produced on average 0.3 log-unit lower inhibitory potencies when compared to the fluorescent substrate uptake assay, which was found to be significant (*p* = 0.016, paired two-tailed Student’s t-test, Table 2). Taken together, these results indicate that the TRACT assay is a suitable method to characterize and screen potential NET inhibitors.

**Table 2:** Inhibitory potency (pIC_50_) values of NET inhibitors determined in the TRACT assay and fluorescent substrate uptake assay.

Inhibitor	pIC_50_^a^			
	TRACT assay^b^	*n*	Fluorescent substrate uptake assay^c^	*n*
Amitriptyline	6.7 ± 0.1	4	7.2 ± 0.1	5
Atomoxetine	7.7 ± 0.1	4	8.6 ± 0.0	3
Benztropine	6.5 ± 0.1	3	6.1 ± 0.1	3
Bupropion	4.9 ± 0.1	3	5.5 ± 0.0	3
Cocaine	6.2 ± 0.1	4	n.d.^d^	
Desipramine	8.2 ± 0.1	3	8.6 ± 0.1	5
GBR12909	6.1 ± 0.2	3	6.0 ± 0.1	3
Maprotiline	7.3 ± 0.1	4	7.4 ± 0.1	3
Milnacipran	7.3 ± 0.2	4	7.8 ± 0.0	3
Nisoxetine	8.3 ± 0.1	7	8.4 ± 0.1	5
Nortriptyline	7.4 ± 0.1	5	8.0 ± 0.1	5
Reboxetine	8.3 ± 0.1	5	8.8 ± 0.0	5

^a^The mean pIC_50_ values found in the TRACT assay were on average 0.3 log-units lower than in the fluorescent substrate uptake assay (*p* = 0.016, paired two-tailed Student’s t-test).

^b^In the TRACT assay, values are reported as the mean ± SEM of three to seven individual experiments performed in duplicate.

^c^In the fluorescent substrate uptake assay the values are reported as the mean ± SEM of three to five individual experiments each performed in quadruplicate.

^d^Not determined.

## Discussion

The functional characterization of inhibitors for neurotransmitter transporters, such as NET, is conventionally done by performing radioligand or fluorescent substrate uptake assays, which can also be used to derive kinetic parameters (K_m_, V_max_) of substrates for a specific transporter^15,17,18^. However, radioligand uptake assays are generally labor-intensive, end-point measurements and restricted to low-throughput screening due to practical limitations in handling of radioactive materials^15,19^. Fluorescent substrate uptake assays, on the other hand, overcome these limitations by allowing one-step, real-time measurements in live cells and have the potential for high-throughput screens^17,18,31^. Despite this, a fluorescent substrate first needs to be designed, synthesized and thoroughly validated. Moreover, the chemical modification of a substrate in order to generate a fluorescent readout could influence the observed response when regarding the native substrate(s) of the transporter^32^. The TRACT assay presented in the current study demonstrates that GPCR activation can be used as a readout to infer NET transport function, allowing functional characterization of NET inhibitors in live cells using unmodified, endogenous substrates.

The TRACT assay principle assumes that the substrate of the transporter is able to induce a cellular response (e.g., by activation of a cell surface GPCR), where the transporter activity (i.e., uptake of substrate) indirectly affects the magnitude of the substrate-induced response, as has been shown recently^20^. Besides its main substrate, NE, NET is known to transport the catecholamines DA and EP^33,34^ as well as other amines and substances such as tyramine, phenylethylamine and MPP+^6^. Since NE, DA and EP are reported endogenous agonists for adrenergic and/or dopamine GPCRs^35–37^, it was hypothesized that these could be used as substrates in the TRACT assay. Indeed, all three substrates were able to induce concentration-dependent cellular responses in JumpIn-NET cells (Figs. 1, 2, 3), which were mainly attributed to the activation of α_2_ARs (Fig. 4). Cellular responses to these catecholamines in unmodified HEK293 cells have previously been observed using a label-free optical biosensor, indicating that adrenergic receptors are commonly expressed in these cells^38^. Notably, a comparable observation was made recently in the TRACT assay for the dopamine transporter (DAT) using a similar JumpIn cell line, in which the substrate DA activated α_2_ARs^22^.

The suitability of each substrate to measure NET activity was dependent on the apparent potency of the substrate for the GPCR. In the TRACT assay the apparent potency of DA was not significantly increased by nisoxetine in the presence of NET (Fig. 2d), most likely due to the poor potency of DA on α_2_ARs. A possible alternative to increase the substrate sensitivity and inhibitory assay window could be to co-express a high affinity dopamine receptor (e.g., D_1_ or D_2_ dopamine receptor^22,39^). This could lead to a more leftward-shifted concentration–response curve in cells lacking NET (−dox) or cells expressing NET (+dox) in presence of a NET inhibitor. In our previous TRACT assay for DAT, U2OS cells with endogenous D_1_ receptor expression displayed a slightly higher DA potency and shift compared to α_2_AR-expressing JumpIn-DAT cells^22^, although this might be further improved by heterologous expression of high affinity receptors. This matching of transporter substrate and receptor potency could be optimized for each TRACT assay.

When using NE and EP in the TRACT assay a rapid, transient negative nCI peak was observed upon substrate addition, which was likely related to activation of beta adrenergic receptors on the JumpIn cells (Fig. 4a, c). However, this part of the substrate response was not affected in the presence of a NET inhibitor nor did it substantially contribute to the overall AUC. This indicates that a complex impedance signal comprised of more than one (GPCR) signaling event can be used to define a TRACT assay window^40^. Although receptor activation provides a sensitive readout in this assay, caution is warranted when interpreting the data since NET inhibitors could potentially display activity at the same (adrenergic) receptors^41^. If a compound would be a receptor agonist as well this could be observed as an impedance change during pretreatment, whereas a receptor antagonist would lead to a reduction rather than enhancement of the substrate-induced response. In both cases receptor-related activity would be observed in both cells lacking (−dox) and expressing (+dox) NET, whereas selective NET inhibitors only display activity in NET-expressing cells. Most of the inhibitors that were tested in the TRACT assay did not substantially affect the impedance during pretreatment, indicating a lack of GPCR-related effects of the inhibitors (Supplementary Fig. S1). A transient increase in nCI was observed at the highest tested concentration (10 µM) of atomoxetine, benztropine, bupropion, maprotiline and nisoxetine, although these impedance changes could not directly be attributed to receptor activation or other off-target effects. To correct for any inhibitor-induced impedance changes during the pretreatment the CI data was normalized prior to substrate (NE) addition.

While all three substrates induced GPCR-mediated cellular responses, differences were observed between the substrates regarding concentration-effect curves of non-induced and dox-induced cells (Figs. 1, 2, 3, Table 1). Interestingly, dox-treated cells produced a considerable increase in steepness of the pseudo-Hill slope for NE (n_H_ = 2.1 ± 0.2) and DA (n_H_ = 1.5 ± 0.1), but not for EP (n_H_ = 0.8 ± 0.1), which is in line with our recent observation of increased slopes of DA in cells expressing DAT^22^. This finding fits with previously reported pharmacological experiments on innervated nictitating membranes (expressing NET) of pithed cats, where it was demonstrated that concentration-effect curves of sympathomimetic amines (e.g., norepinephrine, epinephrine) were steeper and right-shifted compared to membranes in the presence of cocaine or in denervated membranes (i.e. lacking NET)^42^. Specifically, the slopes were dependent on the affinity (K_m_) and maximum uptake rate (V_max_) of the substrate, whereas the horizontal curve shift was related to the potency (EC_50_) of the substrate^42,43^. Whereas these findings may in part explain the observed changes in slope and horizontal shift of concentration-effect curves for NE and DA in this study, we could not rationalize the lack of a change in slope for EP. Nevertheless, the steep slope for NE provides a rationale that the largest window for NET inhibition by nisoxetine is found when NE is used as a substrate.

In the present study, it was demonstrated that the TRACT assay can be used to accurately determine inhibitory potency values of NET inhibitors, as a direct comparison to a commercially available fluorescent substrate uptake assay^18^ resulted in similar values that were highly correlated (Fig. 6d). While the rank order of potencies for both assays were comparable, the pIC_50_ values were generally found to be lower in the TRACT assay than in the fluorescent substrate uptake assay (*p* = 0.016, Table 2). A possible reason for this inter-assay discrepancy might be that the assays use a different substrate to determine inhibitory potency, which may affect binding affinity or kinetics of the inhibitor^44^. Although the substrate identity and concentration in the fluorescent substrate uptake assay were not disclosed by the supplier, a reasonable explanation could be that the transporter occupancy by NE is higher than the fluorescent substrate leading to increased inhibitor competition and thus lower pIC_50_ values in the TRACT assay. Alternatively, the uptake process might be rapidly saturated in the TRACT assay in presence of relatively high concentrations of NE, which could lead to an underestimation of the potency of inhibitors^45^. Although information on uptake kinetics could provide a more substantiated explanation to this, a drawback of the TRACT assay is that it cannot be used to directly measure the substrate uptake kinetics (e.g., K_m_, V_max_). Nevertheless, the inhibitory potency values of all tested inhibitors were in line with previously reported values from both fluorescent substrate uptake assays^17,18,46,47^ and radioligand uptake assays^16–18,48–51^ indicating that the TRACT assay can be reliably used for NET inhibitor characterization.

The TRACT assay for NET was validated in a manual HTS set-up to assess the assay robustness. Over a three day period, an average Z’ of 0.55 ± 0.04 was obtained which generally indicates an “excellent assay”^26,30^. This score is comparable to previous high-throughput analyses of fluorescent substrate uptake assays by Jørgensen et al*.* (Z′ = 0.43^18^), Haunsø et al*.* (Z′ = 0.64–0.79^17^) and Wagstaff et al*.* (Z′ = 0.61–0.63^46^). While this Z’ value can be considered acceptable, the overall robustness could be further optimized. For example, standardization of cell and compound handling can improve the overall performance and decrease intra-plate and inter-plate variability^30^. Other considerations for optimization of the assay window and robustness are the consistency in confluence and homogeneity of cells, cell density, inhibitor pretreatment duration, buffer/medium composition and DMSO tolerability (generally < 1% final concentration in live cell assays)^30,52,53^. In this study, three E-plates were manually run per day, which would not be considered “high-throughput” and as such the work-flow should be optimized if the TRACT assay is to be used on a larger scale^54^. For instance, impedance measurements could be taken over time for 30 min (using the AUC for analysis) or impedance can be measured once 30 min after stimulation, effectively making the assay a single-point measurement. With proper automation and plate handling systems, the potential throughput per RTCA station would increase from two E-plates per hour (measurement over time) to approximately 30 E-plates per hour (single-point measurement). In the latter case, the amount of plates that could be run per day (360 plates, assuming a 12-h shift) would compare to the estimated throughput of a FLIPR^TETRA^ system^46^. Scale-up of the assay to a multi-plate xCELLigence station that can hold up to six 96-well E-plates simultaneously, or 384-well E-plate format is also an option, but in all cases adjusting the plate format or data acquisition method would necessitate additional optimization of the assay conditions to ensure a robust assay window.

In summary, this study demonstrates the potential of the recently described TRACT assay to be utilized as a high-throughput screening platform for inhibitors of NET. The inhibitory potencies of several well-known NET inhibitors could be accurately determined and the robustness and reproducibility of the assay was validated. Hence, this work makes a case for the TRACT assay as a viable alternative to conventional uptake assays and underpins the breadth of possibilities of using label-free biosensor technologies in drug discovery.

### Supplementary Information

Supplementary Information.

## Data Availability

The datasets generated during and/or analyzed during the current study are available from the corresponding author on reasonable request.
