# Supplementary material for: Label-free high-throughput screening assay for the identification of norepinephrine transporter (NET/SLC6A2) inhibitors
Source: Sci Rep. 2021 Jun 10;11:12290. doi: 10.1038/s41598-021-91700-7 (PMC8192900; doi:10.1038/s41598-021-91700-7)
Supplement: Supplementary file 1 — Supplementary Information. [file 41598_2021_91700_MOESM1_ESM.pdf]

Label-free high-throughput screening assay for the identification of norepinephrine transporter (NET / SLC6A2) inhibitors

Authors

Hubert J. Sijben [a], Wieke M. van Oostveen [a], Peter B.R. Hartog [a], Laura Stucchi [b], Andrea Rossignoli [b], Giovanna Maresca [b], Lia Scarabottolo [b], Adriaan P. IJzerman [a], Laura H. Heitman [a,c,*]

[a] *Division of Drug Discovery and Safety, LACDR, Leiden University, Leiden, The Netherlands*

[b] *Axxam S.p.A., Openzone Science Park, Bresso, Milan, Italy*

[c] *Oncode Institute, Leiden, The Netherlands*

* **Corresponding author**: Division of Drug Discovery and Safety, LACDR, Leiden University, P.O. Box 9502, 2300RA Leiden, The Netherlands. Tel.: +31 71 527 4558. Email: l.h.heitman@lacdr.leidenuniv.nl

Supplementary Material

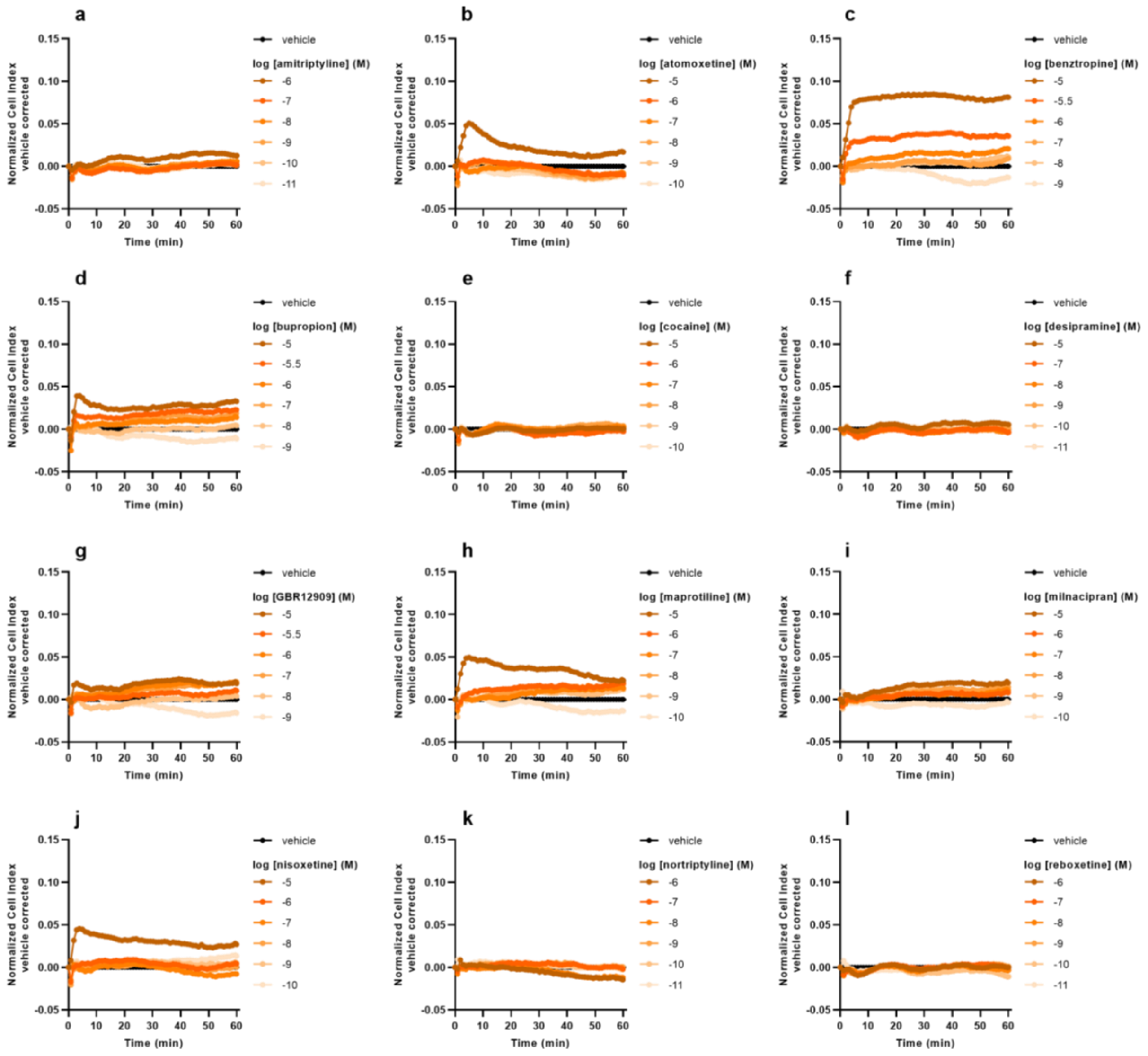

Figure S1. Representative vehicle-corrected xCELLigence traces of NET inhibitors in doxycycline-treated (+dox) JumpIn-NET cells. (**a**) amitriptyline, (**b**) atomoxetine, (**c**) benztropine, (**d**) bupropion, (**e**) cocaine, (**f**) desipramine, (**g**) GBR12909, (**h**) maprotiline, (**i**) milnacipran, (**j**) nisoxetine, (**k**) nortriptyline, (**l**) reboxetine. Graphs show the effect of increasing concentration of the inhibitor on the normalized Cell Index (nCI) during the 1 h pretreatment. Data are normalized to the time point prior to addition of the inhibitor (= 0 min). Data are shown as the mean of a representative graph of at least three separate experiments each performed in duplicate.
